# Role of the ERK Pathway for Oxidant-Induced Parthanatos in Human Lymphocytes

**DOI:** 10.1371/journal.pone.0089646

**Published:** 2014-02-21

**Authors:** Ali A. Akhiani, Olle Werlenius, Johan Aurelius, Charlotta Movitz, Anna Martner, Kristoffer Hellstrand, Fredrik B. Thorén

**Affiliations:** 1 Sahlgrenska Cancer Center, Institute of Biomedicine, The Sahlgrenska Academy, University of Gothenburg, Gothenburg, Sweden; 2 Sahlgrenska Cancer Center, Institute of Medicine, The Sahlgrenska Academy, University of Gothenburg, Gothenburg, Sweden; Southern Medical University, China

## Abstract

Reactive oxygen species (ROS) are formed by myeloid cells as a defense strategy against microorganisms. ROS however also trigger poly(ADP-ribose) polymerase 1- (PARP-1) dependent cell death (parthanatos) in adjacent lymphocytes, which has been forwarded as a mechanism of immune escape in several forms of cancer. The present study assessed the role of mitogen-activated protein kinases (MAPKs), in particular the extracellular signal-regulated kinase (ERK), in ROS-induced signal transduction leading to lymphocyte parthanatos. We report that inhibitors of ERK1/2 phosphorylation upheld natural killer (NK) cell-mediated cytotoxicity under conditions of oxidative stress and rescued NK cells and CD8^+^ T lymphocytes from cell death induced by ROS-producing monocytes. ERK1/2 phosphorylation inhibition also protected lymphocytes from cell death induced by exogenous hydrogen peroxide (H_2_O_2_) and from ROS generated by xanthine oxidase or glucose oxidase. Phosphorylation of ERK1/2 was observed in lymphocytes shortly after exposure to ROS. ROS-generating myeloid cells and exogenous H_2_O_2_ triggered PARP 1-dependent accumulation of poly ADP-ribose (PAR), which was prevented by ERK pathway inhibitors. ERK1/2 phosphorylation was induced by ROS independently of PARP-1. Our findings are suggestive of a role for ERK1/2 in ROS-induced lymphocyte parthanatos, and that the ERK axis may provide a therapeutic target for the protection of lymphocytes against oxidative stress.

## Introduction

The nicotinamide adenine dinucleotide phosphate (NADPH) oxidase transfers electrons from NADPH to molecular oxygen to produce superoxide anion, which is the substrate for a wide range of reactive oxygen species (ROS) including hydrogen peroxide (H_2_O_2_) [1]. Superoxide anion and downstream ROS are produced in the phagosomes of several types of myeloid cells and contribute to the elimination of ingested microorganisms. However, the NADPH oxidase is also present in the plasma membrane, leading to extracellular release of ROS that may damage neighboring cells. In recent years, ROS have been ascribed a role as signaling molecules in immunity, based inter alia on the findings that lymphocyte effector cells such as natural killer (NK) and cytotoxic T cells undergo apoptosis-like cell death after encounter with ROS-producing myeloid cells [2]–[6].

The ROS-induced inactivation of lymphocytes has been implicated in the development of autoimmunity and in cancer-related immunosuppression. Myeloid cell-derived ROS have been ascribed a protective role in autoimmunity by eliminating autoreactive T cells, thus preventing arthritis [7], [8]. In addition, ROS produced by NADPH oxidase-expressing malignant myeloid cells [9], [10] or by tumor-infiltrating macrophages [11]–[18] have been proposed to contribute to the state of NK cell and T cell dysfunction in several forms of cancer, which is the background to the use of ROS inhibitors in cancer immunotherapy [19]–[21]. Defining the intracellular signaling that transduces ROS-induced lymphocyte toxicity may therefore have therapeutic implications.

Recent studies show that ROS-induced cell death in NK and T cells is initiated independently of caspases and instead involves the poly(ADP-ribose) polymerase-1 (PARP-1) nuclear enzyme [4]. PARP-1 is activated by the presence of nicked DNA leading to poly(ADP)-ribosylation of specific acceptor proteins, which recruits enzymes involved in DNA repair [22]. In addition to its role in DNA repair, however, excessive activation of PARP-1 triggers cell death by the release of poly(ADP-ribose) (PAR) fragments into the cytoplasm, which in turn release apoptosis-inducing factor (AIF) from mitochondria followed by DNA fragmentation and apoptosis-like cell death [23]–[26]. The PAR/AIF pathway of cell death was recently termed *parthanatos* to distinguish it from caspase-dependent apoptosis, necrosis and other cell death pathways [27], [28].

ROS are signaling molecules and activate multiple signal transduction pathways, including the phosphorylation cascades leading to the activation of mitogen-activated protein kinases (MAPKs) [29]–[31]. Based on structural differences, MAPKs encompass at least six subfamilies, among which the ERK1/2, JNK, and p38 kinase are the most extensively studied [32]. ERK1/2 is activated by MEK1/2, which is downstream of the Ras/Raf pathway and has been implicated in mitogenesis, cell differentiation, and stress responses [33]. While the specific role of ERK for ROS-induced lymphocyte cell death is not known, ERK1/2 has been implicated in preventing cell injury induced by oxidative stress in HeLa cells and fibroblasts [34], [35]. In contrast ERK activation was reported to contribute to cell death induced by oxidants such as H_2_O_2_ in oligodendrocytes [36], [37], mesangial cells [38], glioma cells [39], neuroectodermal cells [40], and gingival fibroblasts [41].

The present study sought to clarify the role of MAPKs, in particular their relation to the PARP-1 pathway, in the signal transduction leading to ROS-induced cell death in human lymphocytes. Our data are suggestive of a previously undefined molecular link between oxygen radicals, ERK1/2, and PARP-1 of relevance to lymphocyte parthanatos.

## Materials and Methods

### Ethics statement

This study was performed on anonymized buffy coats obtained from the blood bank at the Sahlgrenska University hospital, Gothenburg, Sweden. Since obtained results could not be traced back to a specific individual, ethical approval was not needed according to Swedish legislation (4§, SFS 2003:460).

### Cell samples and isolation

Leukocytes were obtained from freshly prepared acid citrate dextrose-containing leukopacks from healthy blood donors at the Blood Centre (Sahlgrenska University Hospital, Gothenburg, Sweden). The blood was either mixed with equal volumes of phosphate-buffered saline (PBS) or, in some experiments, with 2% dextran followed by incubation for 15 minutes, to remove erythrocytes. The cell suspension or supernatant, respectively, were then carefully layered on top of a Ficoll-Hypaque (Lymphoprep) density gradient. After centrifugation at 850×*g* for 15 min, peripheral blood mononuclear cells (PBMCs) were collected at the interface [9]. PBMCs were washed and further separated into lymphocytes and monocytes using countercurrent centrifugal elutriation as described [2], [42]. A fraction with >96% monocytes (CD14^+^) was obtained along with fractions of enriched NK cells (CD3^−^56^+^ phenotype) and T cells (CD3^+^56^−^ phenotype). In some experiments, CD14^+^ monocytes were negatively enriched from PBMCs using the MACS monocyte isolation kit II (Miltenyi Biotec, Germany) according to the instructions provided by the manufacturer. Notably, this method involves a step in which monocytes are incubated with an Fc-receptor blocking reagent. In these experiments, the purity of isolated monocytes exceeded 92%.

NK cells and CD8^+^ T cells were further enriched from the elutriated lymphocyte fractions or from PBMCs using the MACS NK and the MACS CD8^+^ T cells negative isolation kits (Miltenyi Biotec) according to the manufacturer's instructions. Briefly, undesired cells were magnetically labeled and depleted using a cocktail of biotin-conjugated Abs and magnetic microbeads. The purity of isolated NK cells and CD8^+^ T cells were analyzed by flow cytometry using monoclonal antibodies to CD3, CD8 and CD56. The purity of isolated cells exceeded 96%. Separated cells were resuspended in Iscoves' modified Dulbecco minimum essential medium (IDMEM) supplemented with 10% human AB^+^ serum. This medium was used in all experiments.

### Cell death

The purified NK cells or CD8^+^ T cells were preincubated in 96-well plates with a MEK1/2 inhibitor (25 µM PD98059, Merck, Darmstadt, Germany, 12.5 µM AZD6244, Selleck Chemicals, Houston, TX or 1.6 µM U0126, Sigma-Aldrich, St Louis, MO, USA), or DMSO (0,05%, Sigma-Aldrich) for 1 hr at 37°C before overnight incubation with autologous monocytes or exogenous H_2_O_2_ (Sigma-Aldrich,). In some experiments, NK cells were preincubated with PD98059 (25 µM) followed by overnight incubation with H_2_O_2_ inducers such as xanthine (0.03–0.25 mM, Sigma-Aldrich) plus xanthine oxidase (100 mU/ml, Sigma-Aldrich) or glucose oxidase (1.25–5.0 mU/ml, Sigma-Aldrich). Xanthine oxidase converts exogenously added xanthine to uric acid and H_2_O_2_ [43] and glucose oxidase catalyses the oxidation of glucose present in IDMEM to gluconic acid with the formation of H_2_O_2_. After 16 hours, NK cells and CD8^+^ T cells were harvested from the plates and analyzed using flow cytometry. Loss of structural integrity of the plasma membrane was determined by staining cells with Live/Dead fixable violet dead cell stain (Invitrogen, Carlsbad, CA, USA) for 20 min at 4°C. Cell death in NK cells and CD8^+^ T cells was then assayed using a BD FACSAria III (BD Biosciences) equipped with BD FACS Diva version 6.1.3 software. Cell death was measured as the percentage of the Live/Dead Fixable Violet positive cells and confirmed using the altered light-scattering characteristics displayed by dead cells, i.e., a reduced forward scatter and an increased side scatter signal [2]. PD98059 was initially dissolved in DMSO. The final concentration of DMSO in the tissue culture medium was 0.05%, which did not affect cell viability.

### ADCC assay

NK cells were assayed for antibody-dependent cellular cytotoxicity (ADCC) against cells of the human B lymphoblastoid cell line 721.221 (221) [44] coated with anti-CD20 (rituximab, Roche, Basel, Switzerland). Purified NK cells were first preincubated with either PD98059 (25 µM) or DMSO (0.05%) for 1 hour and subsequently co-incubated with autologous monocytes at monocyte:NK cell ratios of 0.25∶1, 0.5∶1 and 1∶1 respectively. After 16 hours, rituximab 10 μg/ml and CFSE-labeled 221 cells were added at an effector to target cell ratio of 1∶1. After another four hours of incubation the plates were centrifuged at 500G for 5 minutes, the supernatant discarded, and the cells stained with the Live/Dead Fixable Far Red Dead Cell Stain kit (Invitrogen, Carlsbad, USA). Cell death in target cells and NK cells was assessed using a BD LSRFortessa flow cytometer and BD FACS Diva software as described above.

### Measurement of ROS

Potential non-specific effects of inhibitors on superoxide anion production by myeloid cells were determined using isoluminol-enhanced chemiluminescence as described elsewhere [45]. In brief, mononuclear myeloid cells were stimulated by *N*-formyl-methionyl-leucyl-phenylalanine (fMLF, 0.1 µM) in Kreb's Ringer glucose buffer (KRG) supplemented with isoluminol (10 µg/ml) and horseradish peroxidase (HRP, 4 U/ml), and light emission was recorded continuously in a six-channel Berthold Biolumat LB 9505 (Berthold Technologies Co, Wildbad, Germany). Experiments were also performed in which myeloid cells and fMLF were replaced by xanthine/xanthine oxidase to determine the potential ability of inhibitors to neutralize ROS [43], [46]. In addition, the ability of inhibitors to consume hydrogen peroxide was determined as described [5], [45]. In brief, inhibitors were diluted in KRG to concentrations used in cell death experiments and incubated with 50 µM H_2_O_2_ for 15 min. Catalase (200 U/ml) was used as positive control. The remaining H_2_O_2_ was determined fluorometrically by measurement of oxidation of p-hydroxyphenyl acetic acid (PHPA) (0.5 mg/ml) catalyzed by HRP (4 U/ml), using a Perkin-Elmer fluorescence spectrophotometer (LC50) (excitation, 320 nm; emission 400 nm).

### Measurement of phosphorylated ERK1/2 expression and PAR formation by flow cytometry

Freshly isolated PBMCs were treated with H_2_O_2_ (500 µM), PMA (phorbol 12-myristate 13-acetate, Sigma-Aldrich) (50 ng/ml) or PBS at 37°C. In some experiments, PBMCs were pretreated with a MEK1/2 inhibitor (25 µM PD98059), a PARP-1 inhibitor (2 µM PJ34, Sigma-Aldrich), DMSO (0.05%), or medium before exposure to H_2_O_2_ or PBS. In additional experiments, freshly isolated NK cells were pretreated with PD98059 (25 µM), PJ34 (2 µM), DMSO (0.05%), or medium before exposure to monocytes in the presence or absence of PMA (50 nM) or PBS. The PBMCs and NK cells were then washed in cold PBS, fixed with cytofix/cytoperm (BD Biosciences, San Diego, CA) and resuspended in ice-cold methanol (Sigma-Aldrich) (final concentration 90%). After 10 min incubation on ice, the cells were washed in PBS with 5% BSA (Sigma Aldrich) and permeabilized with perm/wash (BD) before staining with mouse anti-phospho ERK1/2 (pT202/pY204) mAb (BD) or mouse anti-poly ADP-ribose mAb (BD) followed by incubation with Alexa fluor-488-conjugated goat anti-mouse (Invitrogen) Ab. Cells were then washed and analyzed for PAR accumulation or phosphorylated ERK1/2 expression by flow cytometry.

### Western blot analysis of phosphorylated ERK1/2 expression and PAR formation

Freshly isolated lymphocytes were incubated with H_2_O_2_, PMA (50 ng/ml) or PBS at 37°C. In some experiments, lymphocytes were pretreated with a MEK inhibitor (25 µM PD98059), a PARP inhibitor (2 µM PJ34), DMSO (0.05%) or medium before exposure to H_2_O_2_ or PBS. To prepare lysates, stimulated cells were disrupted in RIPA buffer (Sigma-Aldrich) containing a protease inhibitor cocktail (Sigma Aldrich). The cell lysate was clarified by centrifugation at 8,000×g for 10 min at 4°C to pellet cell debris. The protein concentration of the cell lysate was determined using the Pierce BCA protein assay kit (Pierce Biotechnology, Rockford). The lysate was then mixed with NuPAGE LDS sample buffer (Invitrogen) and NuPAGE sample reducing agent (Invitrogen), heated at 70°C for 10 min, and resolved on 4–12% NuPAGE Novex Bis-Tris precast gels (Invitrogen). Electrophoresis was conducted at 200 V for 50 min and the proteins were transferred to nitrocellulose membranes using iBlot Gel Transfer Device (Invitrogen). The membranes were blocked with buffered saline containing detergent and Hammersten casein solution (Invitrogen), and then washed with PBS-Tween 20. For detection of phosphorylated ERK (p-ERK), membranes were incubated with rabbit anti phospho-ERK1/2 Ab (Cell Signaling Technology, MA, USA) or rabbit total ERK1/2 (Cell signaling technology) followed by incubation with a HRP-conjugated goat anti-rabbit Ab (DAKO). For PAR detection, membranes were incubated with mouse anti-poly ADP ribose mAb (BD) or mouse anti-β tubulin (Invitrogen) followed by incubation with a HRP-conjugated polyclonal rabbit anti-mouse Ab (Dako, Denmark). The signals were visualized using enhanced chemiluminescence (Chemi-Doc, Bio-Rad) with ImmunoStar HRP detection kit (Bio-Rad, Hercules, CA). The intensities of the bands were quantified using the NIH ImageJ [47] software.

### Statistical analysis

One-way ANOVAs followed by Bonferroni's Multiple Comparison Test were used when comparing multiple groups and the Paired T-Test for single comparisons. P values<0.05 were considered statistically significant. In figures, asterisks are used as follows: *P<0.05, **P<0.01, ***P<0.001. All indicated p-values are two-sided.

## Results

### Role of ERK1/2 in oxygen radical-induced lymphocyte cell death

**Figure 1:**
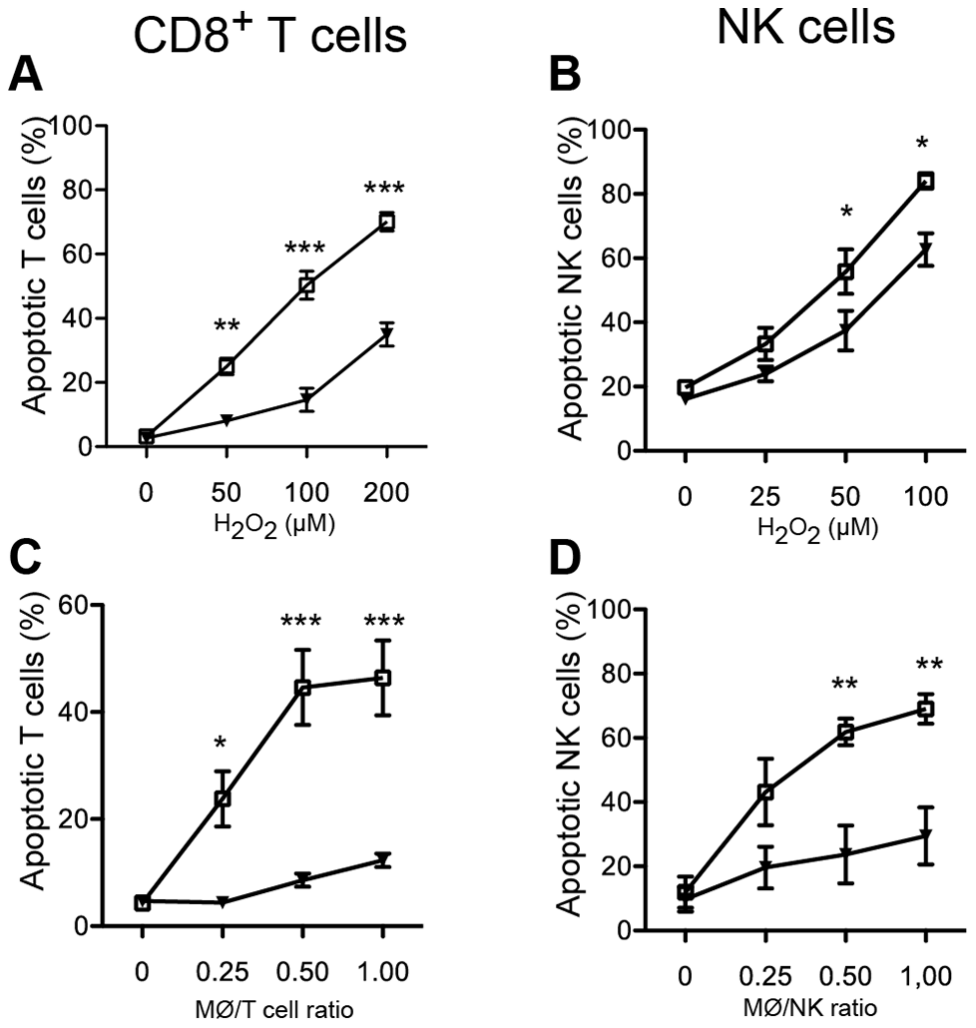
Protection of lymphocytes from ROS-induced apoptosis by an ERK pathway inhibitor. MACS-purified human CD8^+^ T cells or NK cells were preincubated with the ERK1/2 inhibitor PD98059 (25 µM) (filled triangle) for 1 h at 37°C. The T cells and NK cells were then incubated overnight in the presence of PD98059 with H_2_O_2_ at indicated concentrations (**A–B**) or with ROS-producing monocytes (MØ) at indicated MØ:NK ratios (**C–D**). Lymphocyte viability was assessed using the Live/Dead Fixable Violet Dead Cell Stain kit. ERK inhibitor-equivalent concentrations of DMSO were used as control (open square). Results obtained using DMSO did not significantly differ from PBS. Data are the mean ± SEM of results obtained using blood from 3–7 donors. *P<0.05, **P<0.01 and ***P<0.001.

We have previously reported that ROS-induced death of human NK cells and T cells is mediated by the PARP-1/AIF axis [4], but details regarding signal transduction mechanisms upstream of DNA fragmentation and cell death have remained unknown. To elucidate the role of the ERK pathway for these ROS-induced events, lymphocytes were pretreated with inhibitors of the ERK1/2 pathway (PD98059), p38 (SB203580) or JNK (SP600125) before over-night exposure to H_2_O_2_. As shown in figure 1A and 1B, the MEK1/2 inhibitor PD98059, which inhibits phosphorylation of ERK1/2, significantly protected CD8^+^ T cells and NK cells against H_2_O_2_-induced death. These results were confirmed with two other MEK1/2 inhibitors (AZD6244; Figure S1A-B and U0126; Figure S1C–D). SP600125 and SB203580 did not affect H_2_O_2_-induced lymphocyte death (Figure S1E-H).

In additional experiments, oxidative stress was induced in NK cells using the glucose oxidase or xanthine oxidase systems, both of which generate superoxide anion and H_2_O_2_ at a continuous rate in the absence of ROS-producing cells [43], [46]. In accordance with the results obtained with lymphocytes exposed to H_2_O_2_, inhibition of the ERK1/2 pathway but not of JNK or p38 prevented cell death induced by ROS derived from xanthine oxidase or glucose oxidase (Figure S1G and 1H and data not shown).

Human mononuclear myeloid cells (monocytes) isolated from PBMC have been shown to induce parthanatos and down-regulate lymphocyte functions by producing and releasing NADPH oxidase-derived ROS [2], [4]. As shown in figure 1C–D, inhibition of the ERK1/2 pathway efficiently rescued autologous CD8^+^ T cells and NK cells from monocyte-induced, ROS-dependent apoptosis. The striking protection against monocyte-derived ROS may in part be due an effect of ERK 1/2 on ROS or on ROS formation by monocytes. To address this issue, we investigated whether the inhibitors affected ROS formation or neutralized ROS. These experiments showed that the ERK1/2 pathway inhibitors did not significantly affect formation of ROS from monocytes and displayed no significant scavenging activity of oxygen radicals generated by xanthine/xanthine oxidase or by glucose/glucose oxidase as determined by isoluminol-dependent chemiluminescence [45] (Figure S2 and data not shown). Collectively, these data suggest that the protective effect of ERK pathway inhibitors against monocyte-induced cell death in lymphocytes is due to inhibition of the cell death pathway in the lymphocytes, but a non-ROS-related effect on monocytes cannot be formally excluded.

### H_2_O_2_ and monocyte cell-derived ROS activate ERK1/2 in lymphocytes

**Figure 2:**
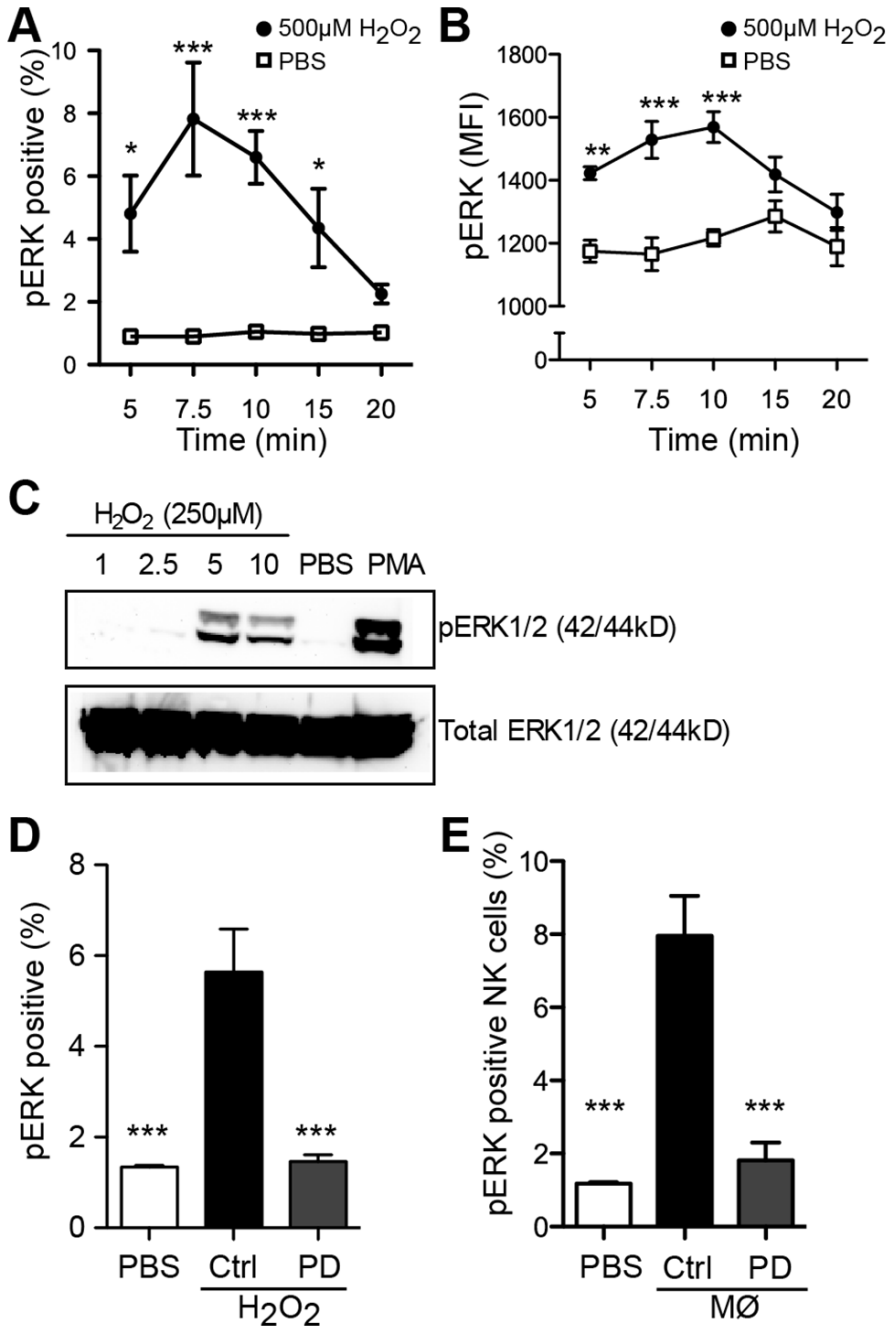
Induction of phosphorylated ERK in ROS-exposed lymphocytes. PBMCs were treated with H_2_O_2_ (500 µM) (filled circle) or PBS (open square) at 37°C and analyzed for pERK by flow cytometry at indicated time points. Graphs show the percentage of pERK-positive cells (**A**) and pERK MFI (**B**) in gated lymphocytes (mean ± SEM, results from 4–6 donors). (**C**) Lymphocytes were treated with 250 µM H_2_O_2_ (1–10 min), PBS (10 min) or 50 ng/ml PMA (40 min) at 37°C. pERK1/2 was detected in whole cell lysates by Western blot. Total ERK1/2 was measured as loading control in parallel wells. A representative blot of 3 is shown. **D.** Inhibition of pERK formation by the ERK1/2 pathway inhibitor (PD98059, 25 µM) in gated lymphocytes after exposure to 500 µM H_2_O_2_ (10 min) shown as percent positive cells. **E.** Inhibition of pERK by PD98059 in NK cells exposed to PMA-stimulated monocytes shown as percent positive cells. Panels D and E are the mean ± SEM of results obtained using 5 donors. *P<0.05, **P<0.01 and ***P<0.001.

To confirm that the observed protection against ROS-induced apoptosis was related to ERK activation, we determined the presence of phosphorylated ERK (pERK) in ROS-exposed lymphocytes. In these experiments H_2_O_2_-treated lymphocytes were fixed, permeabilized, and analyzed for pERK by flow cytometry as described [48]. Figure 2 shows the induction of p-ERK1/2 after ROS exposure with peak activity at approximately 10 min. At each time point a small fraction of lymphocytes stained positively for p-ERK, reflecting the transient nature of ERK phosphorylation induced by oxygen radicals in lymphocytes (Figure 2A), along with an increase in the intensity of p-ERK1/2 on a per cell basis (mean fluorescence intensity, MFI) (Figure 2B). Similar results were obtained using Western blot to detect p-ERK1/2 in lymphocytes exposed to H_2_O_2_ as described [48] (Figure 2C). Phosphorylation of ERK1/2 at 10 min after exposure to H_2_O_2_ was completely inhibited by a ERK pathway (MEK1/2) inhibitor as shown by the percentage of p-ERK1/2 positive cells (Figure 2D) of gated lymphocytes. To clarify whether these events were induced also by a ROS-producing cell, purified NK cells were incubated with autologous monocytes in the presence of the NADPH oxidase activator PMA, which triggers extensive ROS release from monocytes [49]. A robust phosphorylation of ERK1/2 was observed in NK cells 10 min after exposure to the PMA-stimulated monocytes (Figure 2E). The phosphorylation of ERK1/2 in response to monocyte-derived ROS was completely abrogated by the ERK pathway inhibitor PD98059 (Figure 2E).

### Inhibition of the ERK1/2 pathway blocks PAR formation in lymphocytes exposed to H_2_O_2_

**Figure 3:**
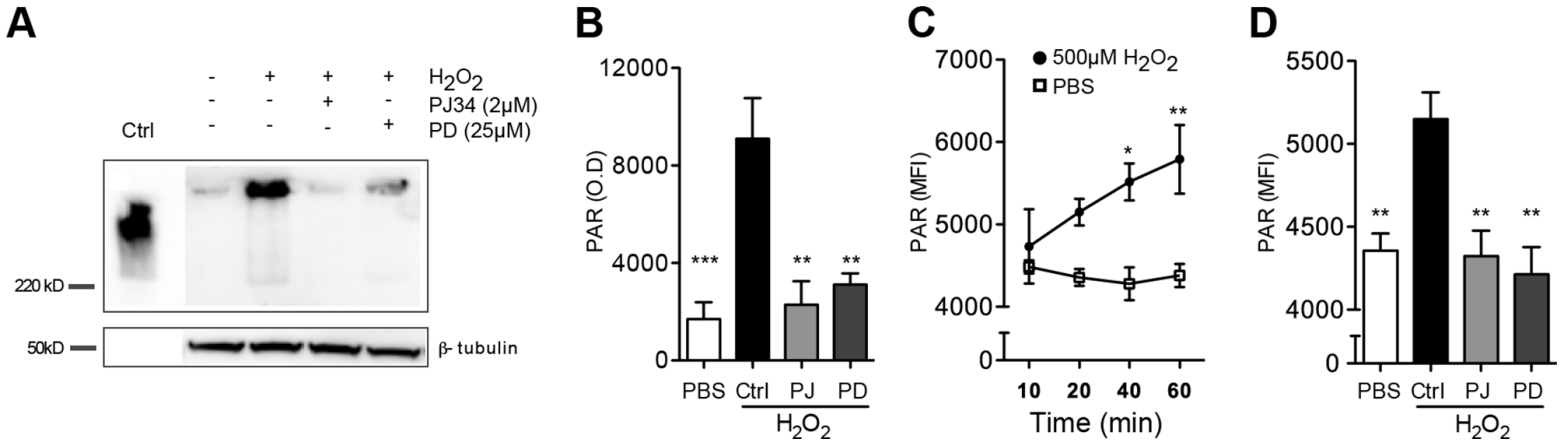
Oxidant-induced poly ADP-ribose accumulation: role of the ERK pathway. (**A–B**) Lymphocytes were preincubated with or without the ERK1/2 inhibitor PD98059 (25 µM) or the PARP-1 inhibitor PJ34 (2 µM) for 1 hr at 37°C before exposure to 500 µM H_2_O_2_ for 20 min. PAR accumulation was analyzed in whole cell lysates. (**A**) Representative Western blot and (**B**) mean ± SEM of results from 4 donors (O.D., optical density). β-tubulin was utilized as loading control and H_2_O_2_-treated HELA cells served as positive controls. (**C**) Flow cytometry analysis of PAR accumulation following exposure to H_2_O_2_ (500 µM, filled circle) or PBS (open square). (**D**) Inhibition of PAR formation in lymphocytes after preincubation with PD98059 (25 µM) or PJ34 (2 µM). PAR accumulation was measured after 20 min exposure to 500 µM H_2_O_2_ by flow cytometry. Data are the MFI of PAR in gated lymphocytes (mean ± SEM of results obtained in 4–6 donors) *P<0.05, **P<0.01 and ***P<0.001).

In a next series of experiments we aimed to clarify a putative link between the ERK and PARP-1 pathways. Lymphocytes were exposed to H_2_O_2_ and the formation of PAR was determined in the presence or absence of an ERK1/2 pathway inhibitor or a PARP-1 inhibitor. As shown in figure 3A and 3B, H_2_O_2_ induced accumulation of PAR in lymphocytes as determined by immunoblotting. The PAR accumulation was prevented by PJ34 and by PD98059. Similar results were obtained with intracellular staining of PAR in lymphocytes using flow cytometry (Figure 3C–D).

### ERK1/2 activation is upstream of PARP-1 in oxidant-induced lymphocyte parthanatos

**Figure 4:**
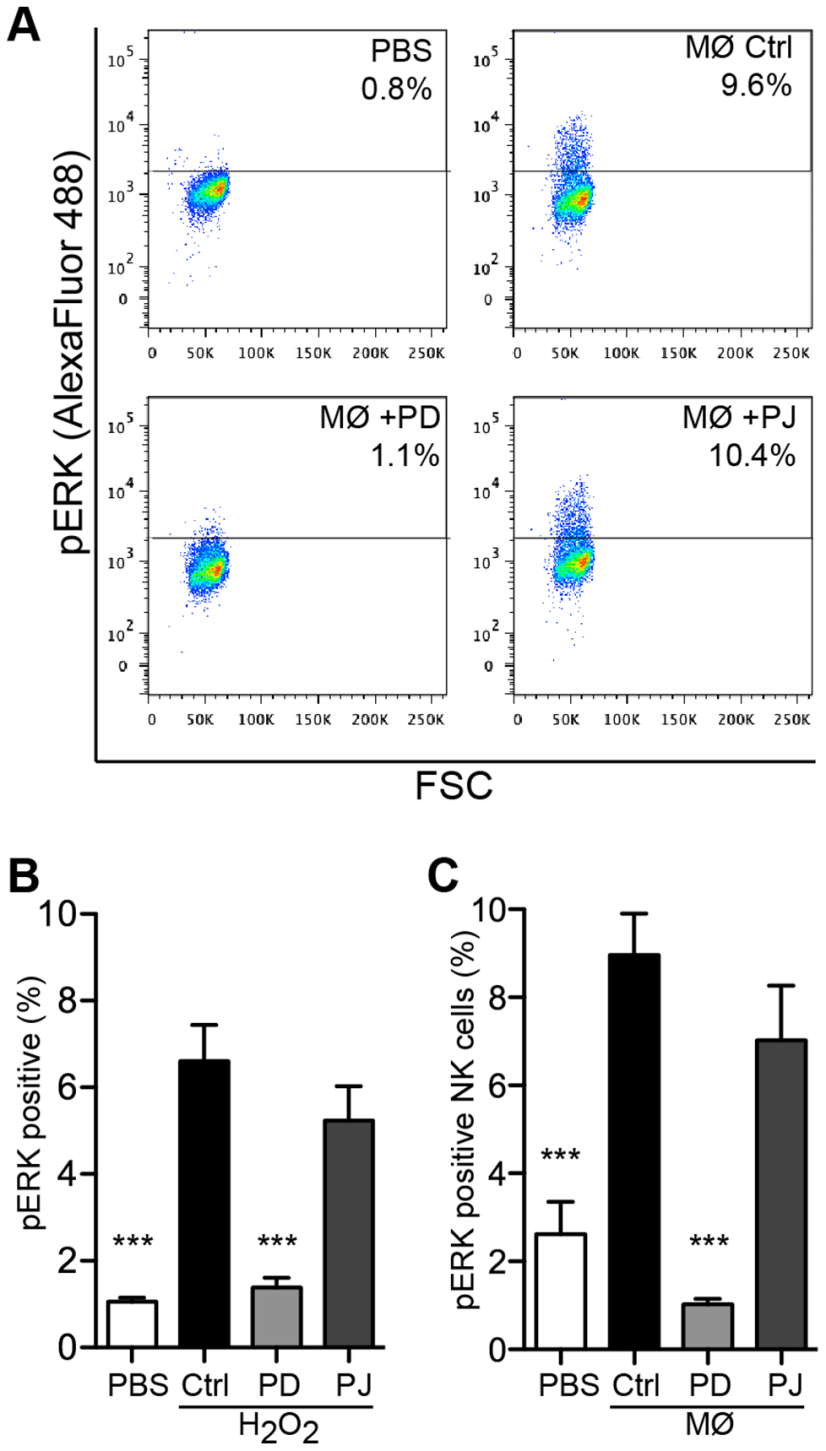
Inhibition of ERK phosphorylation in lymphocytes exposed to oxygen radicals by ERK pathway inhibitor but not by PARP inhibitor. PBMCs or NK cells were preincubated in presence or absence of ERK1/2 inhibitor PD98059 (25 µM) or PARP-1 inhibitor PJ34 (2 µM) for 1 h at 37°C. (**A**) A Representative dot plots of pERK^+^ NK cells after 10 min exposure to PMA-stimulated monocytes_._is shown. (**B**) Mean ± SEM of pERK positive cells in gated lymphocytes after 10 min exposure to H_2_O_2_. (**C**) Mean ± SEM of pERK positive NK cells after 10 min exposure to PMA-stimulated monocytes. **B–C**: mean ± SEM of 4–6 experiments. *P<0.05, **P<0.01 and ***P<0.001.

To clarify whether PARP-1 activation triggers ERK phosphorylation, lymphocytes were preincubated with PJ34, exposed to H_2_O_2_ and assayed for ERK1/2 activation by flow cytometry. An ERK pathway inhibitor strongly reduced phosphorylation of ERK1/2 in the response to H_2_O_2_ whereas the PARP-1 inhibitor was ineffective (Figure 4B and Figure S3). Similar results, i.e. no PARP 1-dependent effect on ERK phosphorylation, were observed when NK cells were preincubated with PJ34 prior to exposure to ROS-producing myeloid cells (10 min exposure to PMA-stimulated monocytes; Figure 4A and 4C). Notably, ERK phosphorylation in T cells and NK cells by PMA alone (in the absence of monocytes and H_2_O_2_) was not detected until after 30 min (not shown) when ERK phosphorylation induced by ROS had returned to the background level (Figure 2A–B).

### NK cells rescued from oxidative stress by ERK-pathway inhibitors are functional

**Figure 5:**
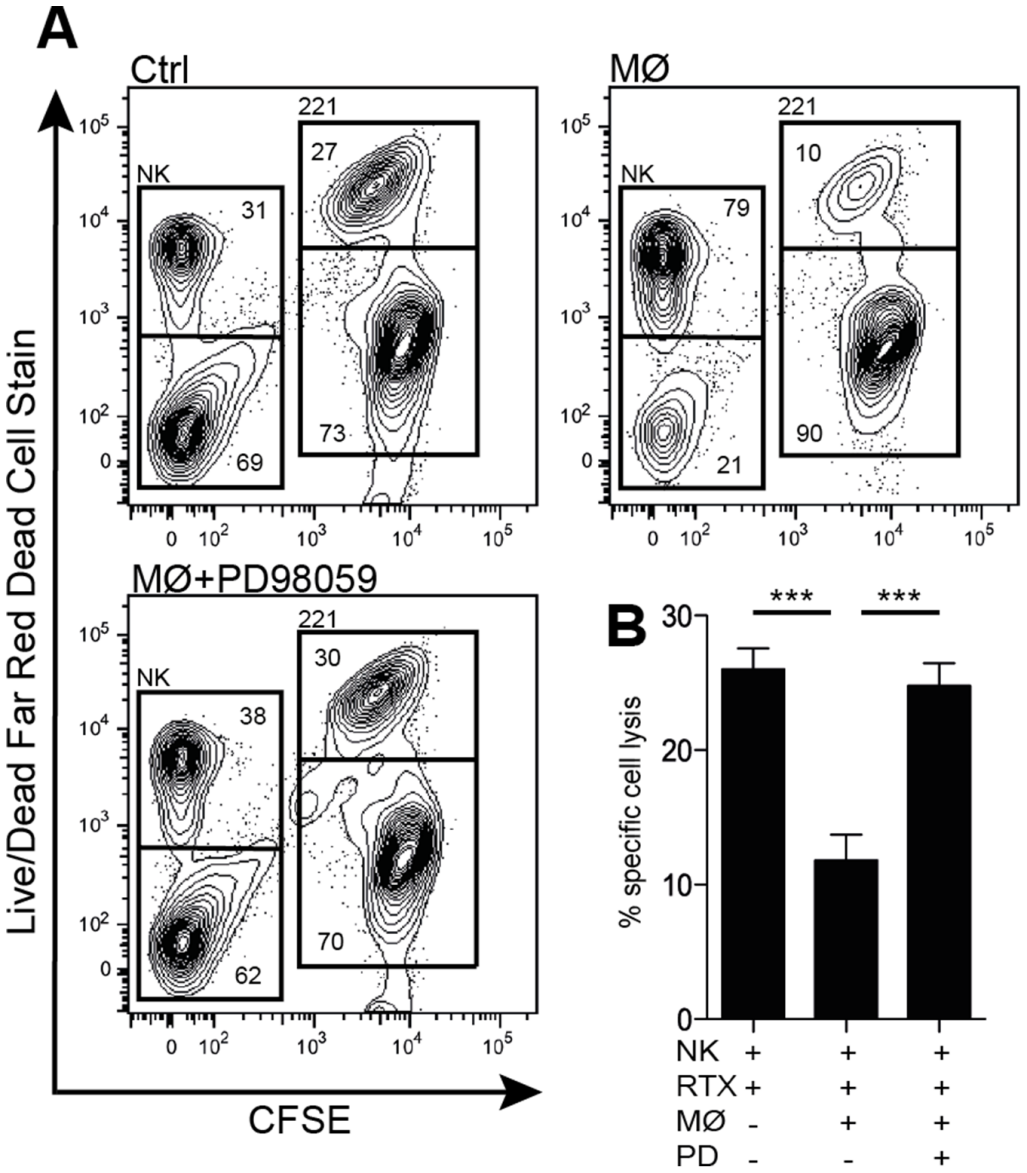
Retained cytotoxicity of NK cells rescued from ROS induced cell death by an ERK1/2 inhibitor. NK cells were co-incubated with monocytes at Mo:NK ratios of 0.25∶1, 0.5∶1 and 1∶1 overnight in the presence or absence of the ERK1/2 inhibitor PD98059 (25 μM). Anti-CD20 (rituximab) and CFSE-labeled 221 target cells were subsequently added at an effector to target cell ratio of 1∶1 as per the NK cell count prior to incubation. Lysis of 221 cells by ADCC was estimated in a 4 hour assay using the Live/Dead Fixable Far Red Dead Cell Stain kit and flow cytometry. Panel (**A**) shows contour density plots from a representative experiment displaying an inverse relationship between NK cell and target cell viability. Panel (**B**) shows NK cell cytotoxicity (mean ± SEM) of seven donors using the lowest Mo:NK cell ratio in which PD98059 rescued NK cells from apoptosis. *P<0.05, **P<0.01 and ***P<0.001.

To investigate whether ERK inhibition of NK cells by PD98059 maintained their functionality after exposure to suppressive myeloid cells, human NK cells were co-cultured over night with monocytes in the presence or absence of PD98059 and subsequently assayed for ADCC against the 221 B-lymphoblastoid cell line. In agreement with earlier studies [50], [51], monocytes, by producing ROS, strongly reduced NK cell cytotoxicity. PD98059 did not affect the ADCC of purified NK cells but significantly upheld NK cell-mediated ADCC in the presence of ROS-producing monocytes (Fig. 5). Similar results were obtained after exposing NK cells to H_2_O_2_ instead of monocytes, (p<0,05, data not shown). Exposure of NK cells to PD98059 did not enhance their ability to induce ADCC, and monocytes did not mediate ADCC against 221 cells under these conditions (Figure S4).

## Discussion

The main findings in this study were that ROS trigger activation of ERK1/2 MAP kinases and that inhibition of the ERK pathway rescues NK cells and T cells from PARP 1-dependent cell death (parthanatos) induced by ROS formed by autologous mononuclear myeloid cells, from ROS generated via xanthine or glucose oxidase and from exogenous ROS (H_2_O_2_). The results thus imply that in human NK cells and T cells, ROS trigger ERK phosphorylation through the MEK signaling pathway, which contributes to parthanatic cell death.

Our study also aimed to define the interplay between ERK1/2 and PARP-1 in the signal transduction leading to parthanatos. PARP-1 is normally involved in DNA strand repair by covalently attaching polymers of PAR to nuclear target proteins, which results in recruitment and activation of DNA repair systems [22], [52]. Upon extensive PARP-1 activation, however, the levels of PAR can transiently increase 10–500-fold [27], [53], which may result in cell death since PAR polymers then translocate from the nucleus to the cytosol with ensuing mitochondrial AIF release and nuclear DNA fragmentation [27]. In accordance with a mode of parthanatic cell death and in agreement with the results of previous report [15], we observed a robust accumulation of PAR in lymphocytes following exposure to H_2_O_2_. ERK1/2 inhibition of lymphocytes exposed to ROS blocked PAR formation and rescued these cells from PARP 1-induced death. In contrast, PARP-1 inhibition did not affect ROS-induced ERK1/2 activation, implying that ERK1/2 is upstream of PARP-1 in ROS-induced lymphocyte parthanatos.

Our results challenge the view that ROS-induced parthanatos is a result of massive DNA damage [27]. In addition to causing DNA damage, ROS play important roles in cell signaling [54], [55]. For example, ROS are known to trigger ERK activation by multiple mechanisms including the activation of upstream receptors, activation of RAS, and inhibition of protein phosphatases (reviewed in [56]. Our data suggest a signaling role for H_2_O_2_ and monocyte-derived ROS, leading to activation of the ERK pathway and subsequent PARP-1 activation. In our study, at each time point after ROS exposure, only a small number of lymphocytes were positive for p-ERK, suggesting that only a transient activation of p-ERK is needed to activate PARP-1. However, MAP kinases have multiple roles and in other studies, ERK 1/2 has been ascribed a role in cell survival [34], [35]. Thus, factors such as the timing and magnitude of the activation, and input from other signaling pathways may all influence whether ERK 1/2 activation promotes cell survival or triggers cell death. The purported link between ERK and PARP-1 that we suggest here is in line with a previous report showing that phosphorylated ERK can activate PARP-1 in the absence of damaged DNA in a cell-free system [57].

In summary, our findings highlight the proposed involvement of ROS in cell signaling [54], [55] and the link between ERK and PARP-1. The results may be of relevance for several forms of cancer, where ROS have been implicated in lymphocyte dysfunction [58]. For example, malignant ROS-producing myeloid cells from patients with acute and chronic myeloid leukemia (AML and CML) were recently reported to induce parthanatos in NK cells [10], [59], [60], which was suggested to contribute to the impaired lymphocyte function in these diseases. In addition, ROS produced by tumor-infiltrating mononuclear myeloid cells have been shown to contribute to lymphocyte dysfunction in several forms of solid cancer [61]. Strategies to protect anti-cancer lymphocytes from inactivation by ROS may therefore be useful in cancer immunotherapies that aim to improve NK cell and T cell functions, as exemplified by the recent introduction of a ROS formation inhibitor, used in conjunction with the NK cell and T cell activating cytokine interleukin-2, for relapse prevention in AML [19], [21]. In the present study we show that NK cells rescued from monocyte ROS-induced cell death by ERK1/2 inhibition retain their capacity to kill lymphoblastoid target cells. Taken together, our results identify the ERK1/2/PARP-1 axis as an additional potential therapeutic target for the preservation of lymphocyte function in cancer immunotherapy. Inhibiting PARP-1 for protection of lymphocytes against oxidative stress inflicted by, e.g., ROS-producing malignant myeloid cells is likely to be limited by the critical role of PARP-1 in DNA-repair. ERK1/2 may thus be a more conceivable target by avoiding detrimental effects on DNA integrity.

## Supporting Information

Figure S1 **Lymphocyte parthanatos and MEK inhibition.** Purified human CD8^+^ T cells (**A, C, E, G and I**) or NK cells (**B, D, F, H and J**) were preincubated with the MEK1/2 inhibitor AZD6244 (12.5 µM) (filled triangle) (**A–B**), MEK1/2 inhibitor U0126 (1.6 µM) (filled triangle) (**C–D**), JNK inhibitor SP600125 (25 µM) (filled triangle) (E–F), the p38 inhibitor SB203580 (25 µM) (filled triangle) (G–H) or equivalent concentration of DMSO (Ctrl, 0.05%, open square) for 1 h at 37°C. Cells were then incubated overnight with H_2_O_2_ at indicated concentrations. Lymphocyte viability was determined using Live/Dead Fixable Violet Dead Cell Stain kit. Data shown are the mean ± SEM of 4–7 experiments. Panels **I and J** show NK cell parthanatos, in presence or absence of PD98059, induced by continuously released H_2_O_2_, generated by xanthine and glucose degradation respectively (mean ± SEM of 5–6 experiments). *P<0.05, **P<0.01 and ***P<0.001.
(TIFF)

Figure S2 **ROS scavenging properties of MAP kinase inhibitors and a PARP-1 inhibitor.** (**A**) The scavenging effect of PD98059 on H_2_O_2_ generated by xanthine oxidase (A) or exogenously added H_2_O_2_ (50 µM) was measured in a cell free system. Briefly, (**A**) xanthine oxidase (10 mU/ml) was allowed to degrade xanthine for 4 minutes in the presence of PBS, DMSO or PD98059. Remaining H_2_O_2_ was measured as chemiluminescence by luminol excitation as described in Materials and Methods. (**B**) PD98059 (25 µM), PJ34 (2 µM) or catalase (200 U/ml) were incubated with H_2_O_2_ (50 µM). After 30 min remaining H_2_O_2_ was assessed as oxidized PHPA, which becomes fluorescent after oxidation. Oxidized PHPA was measured at excitation 320 nm and emission 400 nm using a Perkin-Elmer fluorescence spectrophotometer (LC50). (**C**) The effect of PD98059 on monocyte ROS production was investigated utilizing the luminol system described above. In brief, 5×10^5^ monocytes/ml were incubated with luminol and HRP in the presence or absence of PD98059 or DMSO. ROS production was stimulated with *N*-formyl-methionyl-leucyl-phenylalanine (fMLF, 0.1 µM) or PMA (50 nM) and the chemiluminescence measured continuously over 5 or 20 min respectively. Bars show peak values. A-C. Data shown are mean ± SEM of 3 experiments.
(TIF)

Figure S3 **Increase in MFI in lymphocytes after exposure to H_2_O_2_.** The effect of ERK1/2 pathway inhibitor PD98059 and PARP-1 inhibitor PJ34 on pERK MFI in gated lymphocytes after 10 min exposure to H_2_O_2_ (500 µM). ***P<0.001.
(TIF)

Figure S4 **NK cells, but not monocytes, trigger rituximab-mediated PD98059 insensitive ADCC against 221 cells.** 221 cells were incubated for 4 hours with rituximab (10 µg/ml) and NK cells or monocytes at an E:T ratio of 2∶1, in the presence of either PD98059 (25 µM) or DMSO (0,05%) as control (n = 2).
(TIF)
