# Supplementary figures and images for: Role of the ERK Pathway for Oxidant-Induced Parthanatos in Human Lymphocytes
Source: PLoS One. 2014 Feb 21;9(2):e89646. doi: 10.1371/journal.pone.0089646 (PMC3931820; doi:10.1371/journal.pone.0089646)

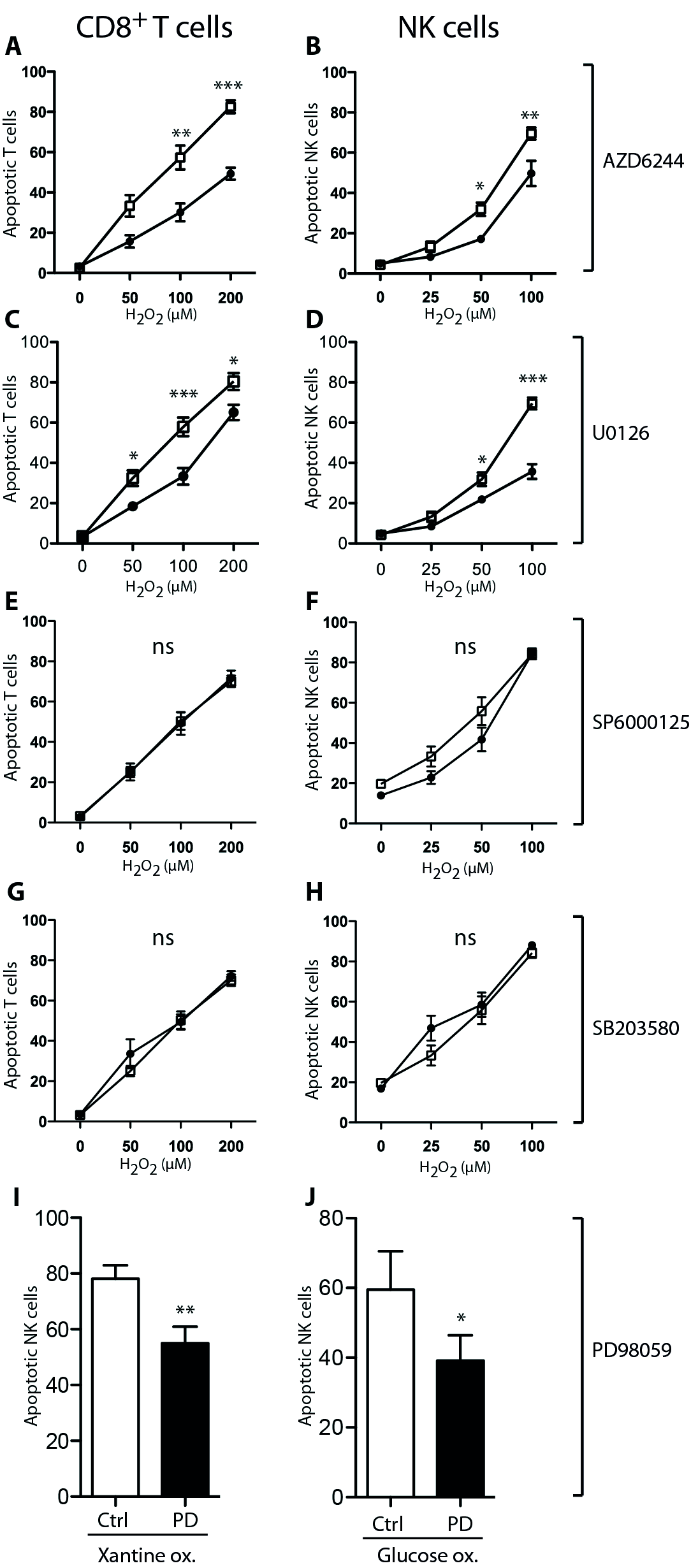

Supplement: Figure S1 — Lymphocyte parthanatos and MEK inhibition. Purified human CD8+ T cells (A, C, E, G and I) or NK cells (B, D, F, H and J) were preincubated with the MEK1/2 inhibitor AZD6244 (12.5 µM) (filled triangle) (A–B), MEK1/2 inhibitor U0126 (1.6 µM) (filled triangle) (C–D), JNK inhibitor SP600125 (25 µM) (filled triangle) (E–F), the p38 inhibitor SB203580 (25 µM) (filled triangle) (G–H) or equivalent concentration of DMSO (Ctrl, 0.05%, open square) for 1 h at 37°C. Cells were then incubated overnight with H2O2 at indicated concentrations. Lymphocyte viability was determined using Live/Dead Fixable Violet Dead Cell Stain kit. Data shown are the mean ± SEM of 4–7 experiments. Panels I and J show NK cell parthanatos, in presence or absence of PD98059, induced by continuously released H2O2, generated by xanthine and glucose degradation respectively (mean ± SEM of 5–6 experiments). *P<0.05, **P<0.01 and ***P<0.001. (TIFF) [file pone.0089646.s001.tiff]

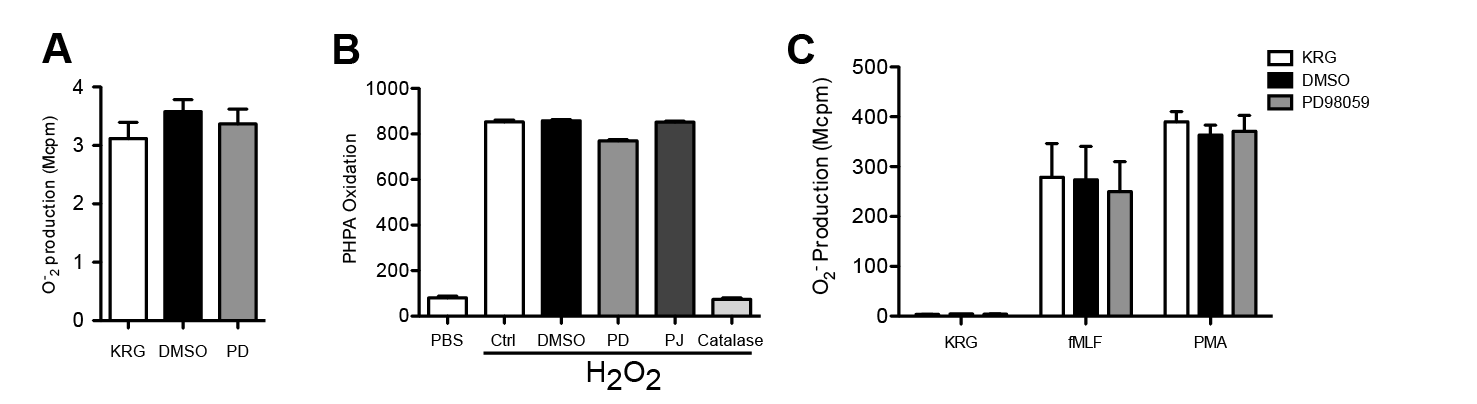

Supplement: Figure S2 — ROS scavenging properties of MAP kinase inhibitors and a PARP-1 inhibitor. (A) The scavenging effect of PD98059 on H2O2 generated by xanthine oxidase (A) or exogenously added H2O2 (50 µM) was measured in a cell free system. Briefly, (A) xanthine oxidase (10 mU/ml) was allowed to degrade xanthine for 4 minutes in the presence of PBS, DMSO or PD98059. Remaining H2O2 was measured as chemiluminescence by luminol excitation as described in Materials and Methods. (B) PD98059 (25 µM), PJ34 (2 µM) or catalase (200 U/ml) were incubated with H2O2 (50 µM). After 30 min remaining H2O2 was assessed as oxidized PHPA, which becomes fluorescent after oxidation. Oxidized PHPA was measured at excitation 320 nm and emission 400 nm using a Perkin-Elmer fluorescence spectrophotometer (LC50). (C) The effect of PD98059 on monocyte ROS production was investigated utilizing the luminol system described above. In brief, 5×10^5^ monocytes/ml were incubated with luminol and HRP in the presence or absence of PD98059 or DMSO. ROS production was stimulated with N-formyl-methionyl-leucyl-phenylalanine (fMLF, 0.1 µM) or PMA (50 nM) and the chemiluminescence measured continuously over 5 or 20 min respectively. Bars show peak values. A-C. Data shown are mean ± SEM of 3 experiments. (TIF) [file pone.0089646.s002.tif]

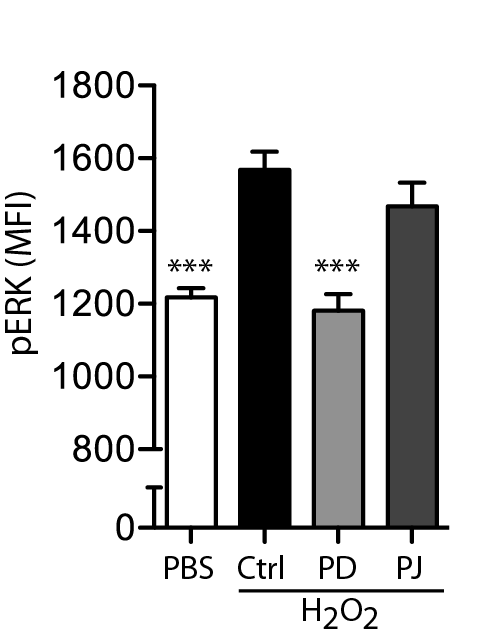

Supplement: Figure S3 — Increase in MFI in lymphocytes after exposure to H2O2. The effect of ERK1/2 pathway inhibitor PD98059 and PARP-1 inhibitor PJ34 on pERK MFI in gated lymphocytes after 10 min exposure to H2O2 (500 µM). ***P<0.001. (TIF) [file pone.0089646.s003.tif]

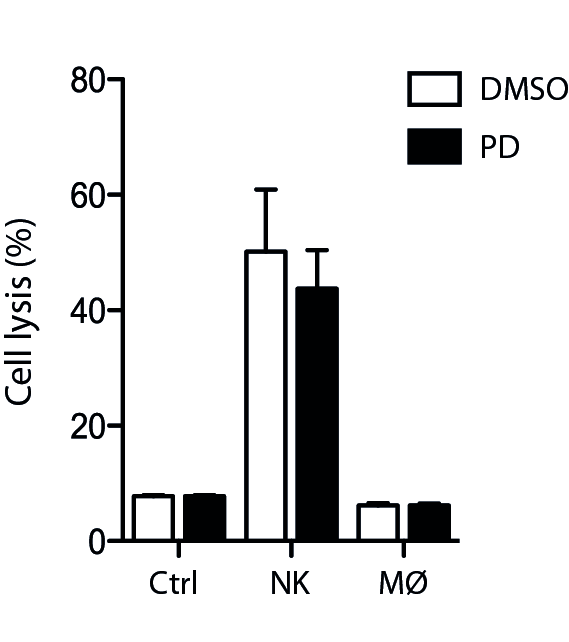

Supplement: Figure S4 — NK cells, but not monocytes, trigger rituximab-mediated PD98059 insensitive ADCC against 221 cells. 221 cells were incubated for 4 hours with rituximab (10 µg/ml) and NK cells or monocytes at an E:T ratio of 2∶1, in the presence of either PD98059 (25 µM) or DMSO (0,05%) as control (n = 2). (TIF) [file pone.0089646.s004.tif]
